# Vulnerability assessment of freeway network considering the probabilities and consequences from a perspective based on network cascade failure

**DOI:** 10.1371/journal.pone.0265260

**Published:** 2022-03-14

**Authors:** Jinqiang Xu, Hainan Huang, Yanqiu Cheng, Kuanmin Chen

**Affiliations:** 1 College of Transportation Engineering, Chang’an University, Xi’an, Shaanxi, China; 2 College of Transportation and Civil Engineering, Fujian Agriculture and Forestry University, Fuzhou, Fujian, China; University at Buffalo, UNITED STATES

## Abstract

Freeway networks are vulnerable to natural disasters and man-made disruptions. The closure of one or more toll stations of the network often causes a sharp decrease in freeway performance. Therefore, measuring the probability and consequences of vulnerability to identify critical parts in the network is crucial for road emergency management. Most existing techniques only measure the consequences of node closure and rarely consider the probability of node closure owing to the lack of an extensive historical database; moreover, they ignore highways outside the study area, which can lead to errors in topological analysis and traffic distribution. Furthermore, the negative effects produced by the operation of freeway tunnels in vulnerability assessment have been neglected. In this study, a framework for freeway vulnerability assessment that considers both the probability and consequences of vulnerability is proposed, based on the perspective of network cascade failure analysis. The cascade failure analysis is conducted using an improved coupled map lattice model, developed by considering the negative effects of tunnels and optimizing the rules of local traffic redistribution. The perturbation threshold and propagation time step of network cascade failure are captured to reflect the probabilities and consequences of vulnerability. A nodal vulnerability index is established based on risk assessment, and a hierarchical clustering method is used to identify the vulnerability classification of critical nodes. The freeway network of Fuzhou in China is utilized to demonstrate the effectiveness of the proposed approach. Specifically, the toll stations in the study area are classified into five clusters of vulnerability: extremely high, high, medium, low, and extremely low. Approximately 31% of the toll stations were classified as the high or extremely high cluster, and three extremely vulnerable freeway sections requiring different precautions were identified. The proposed network vulnerability analysis method provides a new perspective to examine the vulnerability of freeway networks.

## 1. Introduction

A freeway network is the backbone of the intercity transportation network and meets the enormous transportation demand. However, the performance of a freeway network is often disrupted by various types of events. Some events caused by external natural disasters (such as landslides, floods, heavy snow, and earthquakes) may cause extensive damage to the freeway network, whereas others that originate within the transportation system, such as car accidents (especially those occurring in tunnels) and short-term heavy flow, may lead to local road closure and even trigger cascade failure of the network. The cascade of failures is one of the most remarkable processes that propagate in complex networks. It occurs when a failure in a part of the system leads to further failures in the same and other systems in a continuous manner [1]. For example, toll stations in a freeway network may have to be closed because of the sudden occurrence of natural disasters, traffic accidents, or severe traffic congestion. When a toll station is closed, traffic congestion gradually extends upstream. If the adjacent toll stations are close to each other, the upstream extension of traffic congestion is highly likely to trigger a failure of the adjacent toll station. Another more common scenario is that when a toll station fails, the road administrator will immediately take mandatory traffic control measures to direct traffic flow toward the adjacent toll station. The adjacent toll station may then fail owing to this extra traffic load. In this study, a cascade failure implies that the closure of one toll station triggers the closure of the neighboring toll stations. These disruptions have a significant impact on the normal operation of intercity traffic and socio-economic activities. Furthermore, the consequences of interruption on different road sections are significantly different, reflecting the variability of vulnerability. Therefore, it is important to assess the vulnerability and identify the critical parts of the network. Information on the vulnerability of the road network allows road administrators to target emergency preparedness, infrastructure reinforcement, and maintenance procedures to maintain the network’s performance.

Since the concept of vulnerability was first introduced in disaster literature in the 1970s, a number of vulnerability analysis methods have been developed. In the growing and extensive literature on transport vulnerability studies, vulnerability analyses were further classified into topology-based analyses and system-based analyses [2]. A topology-based method has its roots in graph theory and studies the vulnerability of transport networks based on their topological characteristics [3–5]. The method can provide important general insights and indicate various structural weaknesses in transport networks. In contrast, system-based transport vulnerability studies represent the demand and supply sides of transport systems in order to comprehensively assess the consequences of disruptions or disasters for the users and society [6–8]. These studies attempt to overcome some of the limitations of topology-based studies.

Reliability and vulnerability are the most commonly used concepts in studies conducted to evaluate the performance of road networks [9, 10]. They represent the transportation network performance under perturbations from different perspectives. Reliability is measured by the probability that network performance satisfies the required service level under recurrent perturbation, where high probability indicates high reliability; vulnerability is measured as the decrease in network performance under specific non-recurrent perturbation, where a small decrease indicates low vulnerability. In sparse networks such as a freeway network, “vulnerability” of the network can be more important than “reliability” because of the potentially severe adverse consequences of network degradation [11]. The concept of vulnerability is more strongly related to the consequences of link failure; however, it should be noted that the probability of link failure cannot be ignored, because some links in the road network are more prone to failure than others. Examples of such links are links with a defective geometric design, high fluctuations of short-term travel flow, and a large number of long tunnels or tunnel groups, which make the freeways more prone to traffic congestion or accidents that trigger link closures. Even if the consequences of link closures are not too severe, frequent closures of specific links can significantly deteriorate the performance of the road network.

Considering these, some studies have attempted to develop a more comprehensive approach to vulnerability analysis based on a risk analysis perspective [2, 12–15]. In these studies, risk is commonly defined as the product of consequence and probability [11, 13]. The probabilities should be represented by a multivariate discrete–continuous distribution function to assess the relative probabilities of different scenarios [15]. The techniques of hazard identification, risk assessment, and risk evaluation were employed to analyze the risk of closure of the Desert Road, where the risk of closure is the product of the probability of closure and the economic cost of closure [16]. However, determining these probabilities is still an inherently difficult problem as the scale, impact, frequency, and predictability of these perturbation events vary considerably [17]. Among the existing studies on reliability and vulnerability, there have been a few attempts to estimate the probability of disruptions. These include 1) subjective methods such as expert opinion and advice, and community consultation [18, 19]; 2) inventory-based risk assessment, concerned with the state of operation of the various elements of the network (generally its physical components and operating systems) and the likely effects of internal and external factors on the ability of the components to continue functioning [20]; and 3) other methods, such as a stochastic simulation (Monte Carlo-based) method [21], the Monte Carlo simulation method [16], and game theory [22, 23]. The first category of methods is highly subjective and the results are often imprecise. The second and third categories of methods require an extensive and comprehensive historical database, which may not always be available. There is an urgent need to find an alternative method to determine the probability and consequences of vulnerability more conveniently.

In addition to the mathematical assessment models of road network vulnerability, investigators have focused on vulnerability metrics, i.e., the impact factors of vulnerability. Some topology-based metrics have been tested such as the node-degree driven metric [24], node-betweenness and strength [25], shortest path [26], and connectivity. Other metrics based on transportation attributes have also been applied to measure the vulnerability of road networks. These metrics include travel cost, link capacity, flow, and traffic congestion density [27, 28]. Meanwhile, some indicators based on the physical properties of the infrastructure such as the road grade, lane length, and number of lanes have been employed in the vulnerability assessment [29]. However, the impact of tunnels on road network vulnerability is rarely mentioned in the existing literature, although tunnels are increasingly becoming a critical component of road infrastructure. Disruptions that occur in tunnels (especially extra-long tunnels) are often more disruptive to the network due to the lack of adequate emergency response and evacuation capabilities. Ignoring the impact of tunnels may lead to underestimation of the network vulnerability.

Furthermore, there are two issues that need to be fixed in the process of road network vulnerability assessment. The first issue is a possible error that arises from ignoring the border effects in the process of the road network topology representation. Unlike the subway network or the bus network, which has a definite network boundary, the freeway network is usually huge, open, and borderless. The research on freeway networks is usually limited to specific boundaries due to the size limitation of the research area. However, in existing research, the roads or toll stations outside the research boundary of freeway networks are removed subjectively [30], thus causing border effects. These border effects can lead to some topological and flow-distribution errors during network analysis. To the best of our knowledge, none of the published studies appear to account for the effect of border effects when mapping road networks. The other issue is the additional computational burden imposed by a global traffic redistribution across the entire network after link closures. Traffic needs to detour when a link closes. Most of the previous studies have used a user equilibrium model for global road network redistribution after link closures [31, 32]. In addition to increasing the computational burden, this existing method does not correspond to the actual situation because only traffic around the closed link needs to detour when an unexpected disruption event occurs. Therefore, it is necessary to improve the traffic redistribution method for link closures.

To address the problems discussed above, this study attempts to provide an alternative method to obtain the probability and consequences of vulnerability by using cascade failure analysis and proposes a vulnerability assessment framework based on risk analysis. The CML model is improved by refining both the topological and flow coupling coefficients. The topological coupling coefficient considers the negative impact of freeway tunnels on segment operations, and the flow redistribution algorithm is integrated into the flow coupling coefficient. This improved model is then employed to perform a cascade failure analysis of the freeway network to capture both the probability and consequences of vulnerability. In addition, virtual peripheral nodes are introduced to describe scenarios where there is a path extending outside the study area, thus eliminating border effects. This framework eliminates the need for an extensive historical database and facilitates a more comprehensive and convenient vulnerability assessment of the freeway network. By modifying the relevant input parameters of the model, the method proposed in this study can theoretically be applied to the vulnerability and risk analysis of other infrastructure networks.

The remainder of the paper is organized as follows. Section 2 presents the framework of the research method and describes its various stages in detail. Section 3 applies the model to Fuzhou freeway network case studies to demonstrate how the results can be used to support decision making. Finally, conclusions and future prospects are discussed in Section 4.

## 2. Methodology

### 2.1. Vulnerability assessment framework

**Fig 1:**
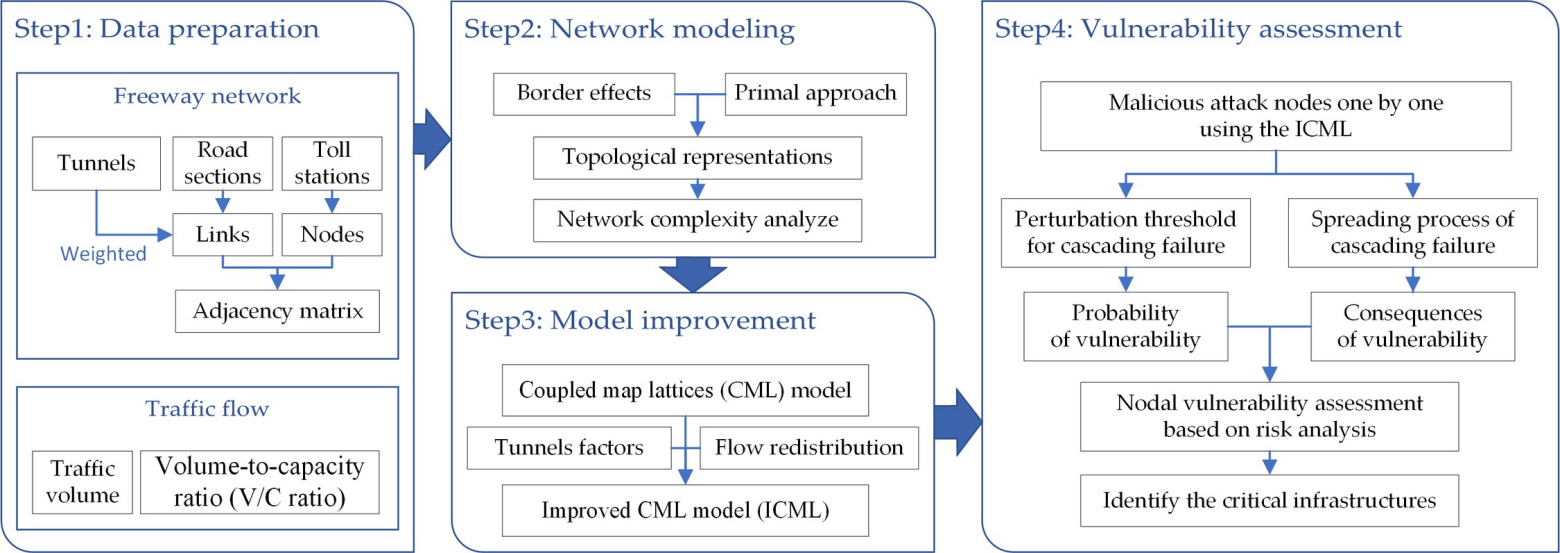
Flow diagram for vulnerability assessment.

The procedure for vulnerability assessment of the freeway network is described in Fig 1. Four steps are involved: First, the basic data of the highway network are collected, including the data of road network and traffic, such as road sections, toll stations, tunnels, traffic volume, etc. Second, the topological structure model of freeway network is established in the primal approach based on consideration of border effects and then the complexity parameters (i.e., degree distribution and average path length) are analyzed. Third, after adding two coupling factors (including tunnel factor and traffic redistribution factor) to the traditional coupled map lattice (CML) model, an improved CML model (ICML) was constructed. Finally, the ICML model is solved in Python to analyze the cascading failures in the case of a malicious attack as a new way to obtain the probability and consequences of vulnerability. The nodal vulnerability is then assessed based on the risk analysis and the critical nodes of the road network are identified using a hierarchical cluster algorithm.

### 2.2. Rules of topological representations considering border effects

According to graph theory [33], the use of reasonable rules for topological representations of a real road network is critical in the analysis of the road network topological structure. Owing to the controlled access and the strict management of the freeway network, the toll stations are the only vehicular entrances and exits of this network. The use of the primal approach is an excellent choice for the representation of the freeway network’s topology [34]. In this study, the freeway network was represented in the form of a directed graph. The freeway toll stations were represented as the network nodes and the freeway section between toll stations as links. The freeway network also contains numerous merging points for two freeways. These merging points are different from the toll stations. In controlled-access freeways, all vehicles can enter and leave the freeway only via special ramps at the toll stations. In contrast, there are no ramps to allow vehicles to enter or leave the freeway network at the merging point of two freeways. The merging point of two freeways has the effect of generating the merging and divergence of traffic in the network. This phenomenon will result in “zero-sum” changes in the traffic volume on the freeway section connected to the merging point but no changes in the total traffic volume. We use the average of the traffic volumes upstream and downstream of the merging point as the weight of the edge containing the merging point. This approach captures the change in traffic volumes upstream and downstream of the merging points. In contrast, from an emergency management perspective, road administrators can respond to emergencies by controlling the number of open lanes at the toll stations. However, road administrators cannot control traffic at the merging points. Therefore, we have not represented these merging points as nodes in the network mapping process for the highway network.

As discussed above, the border effect of the highway network derived from the limitation of the research boundary can lead to errors in topology analysis and flow distribution. Therefore, the virtual peripheral nodes are introduced to eliminate border effects. If a node within the research boundary has a path that extends outside the research area, we must add a virtual peripheral node to connect to this node. If some nodes are indeed the endpoints of roads in the freeway network, no virtual peripheral nodes are added. Peripheral virtual nodes will play a key role in the process of network topology analysis and traffic redistribution.

**Fig 2:**
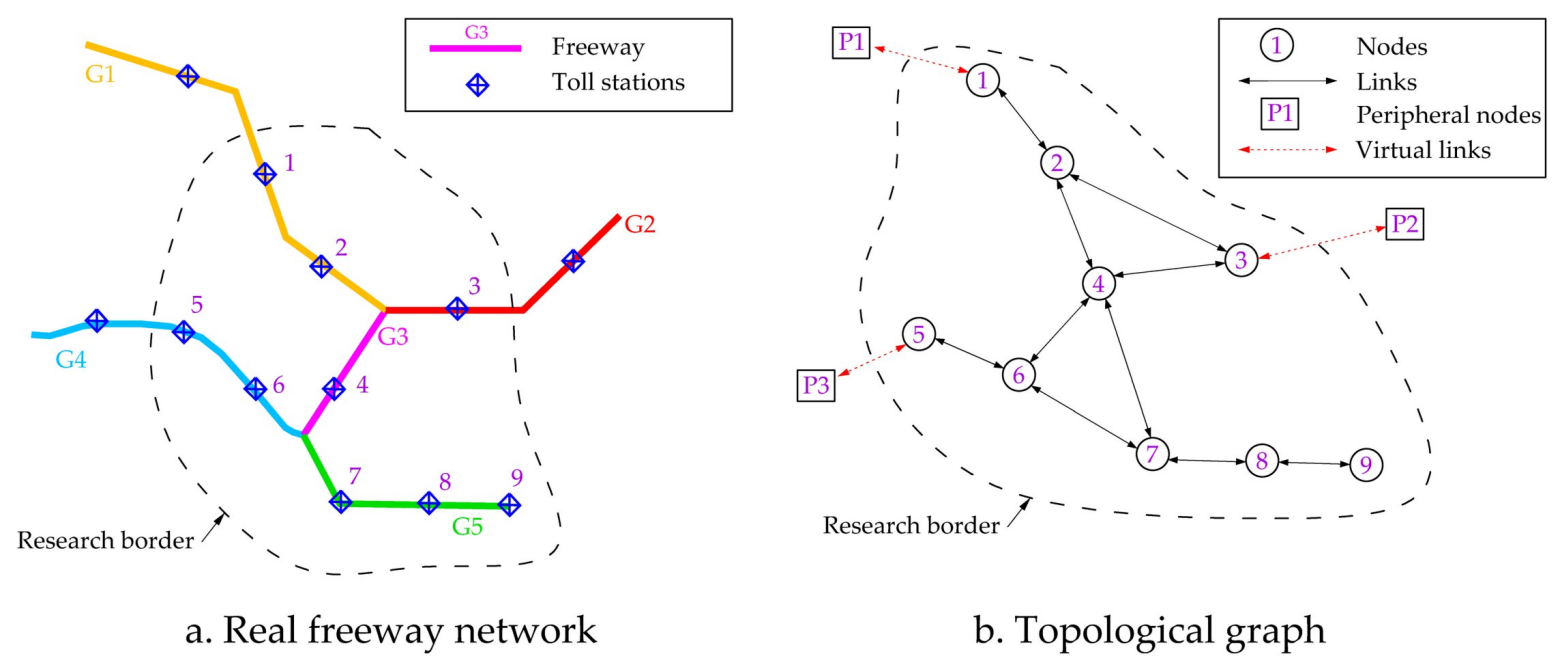
Examples of topological representations considering border effects.

An example of the topological representations for the freeway network is shown in Fig 2. There are five freeways and nine toll stations in the research area (Fig 2(A)). After the topological representation, there are nine nodes and 20 directed links, and there are three virtual peripheral nodes and six virtual links outside the research area (Fig 2(B)). Toll station No. 9 is the endpoint of freeway G5. Thus, there is no virtual peripheral node connected to it.

### 2.3. Cascading failures analysis using improved CML model

#### 2.3.1 Mechanisms of cascading failures and traditional CML model

The failure of a single toll station and the cascading failure of toll stations are two different concepts, and the causes of each are significantly different. The failure of a toll station means that this toll station has to be closed for some reasons. These reasons include natural disasters, traffic accidents, traffic congestion, or short-term heavy flow. However, the cascading failure of toll stations means that the failure of a toll station leads to further failures of neighboring toll stations in a continuous manner. Two common reasons for cascading failures of toll stations are the upstream extension of traffic congestion and mandatory guidance of traffic detours implemented by road managers. Traffic congestion extending upstream is the most direct cause of cascade failure; however, this generally occurs only between two toll stations that are very close to each other. Another more common scenario is that when one toll station fails, the road manager immediately takes mandatory traffic control measures to divert traffic flow to the adjacent toll station. It is highly likely that this extra traffic load will also trigger a failure at the adjacent toll station. Traffic flow then needs to be diverted again to the adjacent toll station, causing a cascading failure. This forced detour guidance of congested traffic flow is more likely to cause failures at adjacent toll stations than gradual extension of traffic congestion upstream.

In the case where multiple toll stations fail simultaneously owing to a natural disaster, the simultaneous failures of these toll stations are not due to cascading failures, but simply multiple “single failures” occurring simultaneously. Of course, such simultaneous failures may also trigger more toll station cascade failures. In this study, cascade failures were investigated under the condition that only one toll station fails initially, without considering how cascade failures occur when multiple toll stations simultaneously fail initially.

The mechanism of cascading failures in this study is significantly different from the conventional spread of traffic congestion. Therefore, conventional traffic flow models, such as the Cell Transmission Model, cannot be employed in this study. In 1992, Kaneko proposed the CML model to study spatiotemporal chaos. CML is a dynamical system that models nonlinear system behavior with the use of partial differential equations [35]. Time and space are discretized variables, and the state is a continuous variable in CML. They are extensively used to qualitatively study the chaotic dynamics of spatially extended systems, including population [36, 37], fluid flow [38], chemical reactions [39], and biological [40, 41] and transportation networks [25, 42, 43]. CML is one of the most widely used methods for studying cascade faults, as it provides a good description of the cascade failure phenomenon [42, 44]. CML has a clear definition of a node’s failure, i.e., a node fails when its state is greater than 1. When a node fails due to a natural disaster, traffic accident, or traffic congestion, this failure is reproduced in the CML by simply adding a perturbation greater than 1 to this node. In addition, the coupling factors in the CML can be increased flexibly according to the characteristics of the study object in order to make the model more relevant and improve its applicability. Therefore, we have used the CML model in this study.

The traditional CML model of *N* nodes is described based on Eq (1) [45].

$$x_{i}(t+1)=|(1-\varepsilon )f(x_{i}(t))+\varepsilon \sum_{j=1\&j\neq i}^{N}a_{i,j}\frac{f(x_{j}(t))}{k(i)}|,\;i=1,2,\cdots ,N,$$ (1)

where *x*_*i*_(*t*) is the state variable of the node *i* at the *t*th time step. The adjacency matrix *A* = (*a*_*ij*_)_*N*×*N*_ represents the topology of the network. If there is a direct connection between nodes *i* and *j*, then *a*_*ij*_ = *a*_*ji*_ = 1; otherwise, *a*_*ij*_ = *a*_*ji*_ = 0. *k*(*i*) is the degree of node *i*, and *ε*∈(0,1) represents the coupling strength. The function *f* defines the local dynamics chosen in this work as the chaotic logistic map, *f*(*x*) = 4*x*(1−*x*). The notation of the absolute value in Eq (1) guarantees that each node’s state is always nonnegative.

The current study of cascading failure in freeway networks focuses on the topological network in CML. However, the cause for cascading failure is not only related to the network topology but also to the flow distribution [25]. Given the freeway network’s characteristics and operating status, two coupling coefficients are considered based on the traditional CML model. Additionally, *ξ*_1_ denotes the topological structure coupling coefficients, and *ξ*_2_ represents the flow coupling coefficients; *ξ*_1_ and *ξ*_2_ are subject to the constraints such that *ξ*_1_, *ξ*_2_ ∈ (0, 1), *ξ*_1_ + *ξ*_2_ < 1.

#### 2.3.2. Topological coupling coefficient *ξ*_1_ based on tunnel factor considerations

The topological analysis in CML is mainly based on the connection relationship of nodes, and degree is a commonly used coupling parameter in CML. For road networks, the attributes of the road section cannot be ignored. The cross-sectional attributes of controlled access freeways are almost identical, but the spatial distribution of the tunnels in the network is very different. Both the traffic capacity and safety performance in the tunnel are lower than that of the freeway mainline. Therefore, tunnels are an important factor in the efficiency of connectivity between freeway toll stations. The low capacities of the tunnel are partly attributed to the driver’s subconscious behavior of increasing the car-following distance for safety reasons. Empirical data show that the capacity of the freeway tunnel is often lower than that of the freeway mainline by 20%-40% [46, 47]. Tunnel operation safety is another important factor that affects the connection. The security of the tunnel is closely related to its length. Traffic accidents occur more frequently in longer tunnels. This is because drivers easily get fatigued in a long tunnel given the monotonous environment when they drive in it for a long time. Moreover, with the increase in tunnel length, the number of vehicles in the tunnel also increases, thus leading to increased exhaust gas concentration and diminished visibility [48]. Therefore, the length of the tunnel has the largest direct impact on the vulnerability of the freeway network.

Therefore, we propose the concept of tunnel factor for the links, $TF_{ij}^{edge}$, to capture the negative effect of tunnels on freeway operations. It is a concept similar to the traffic impedance of roads, and the larger the value of $TF_{ij}^{edge}$, the greater is the negative effect of the tunnels in this link. $TF_{ij}^{edge}$ is defined to reflect both the reduction in tunnel capacity when compared with the mainline and the negative correlation between the length of the tunnel and safety. $TF_{ij}^{edge}$ is defined as in Eq (2).

$$TF_{ij}^{edge}=\{\begin{array}{cc} {\left(1-0.4\frac{L_{ij}^{T}}{L_{ij}}\right)}^{-1} & j\in v(i)\\ 0 & j\notin v(i) \end{array},$$ (2)

where *L*_*ij*_ is the distance between nodes *i* and *j*. $L_{ij}^{T}$ is the total length of a tunnel at the section between nodes *i* and *j*. If there is no one tunnel between nodes *i* and *j*, then $L_{ij}^{T}=0$ and $TF_{ij}^{edge}=1$, which means that the capacity of this edge is not affected by the tunnel.

The tunnel factor to the nodes is related to the edge connected to it, and is estimated based on Eq (3).

$$TF_{i}^{node}=\sum_{j\in v(i)}TF_{ij}^{edge},$$ (3)

where *n* is the number of the edges connected to node *i*, *v*(*i*) is the set of neighbors of node *i*.

#### 2.3.3. Flow coupling coefficient *ξ*_2_ based on flow redistribution considerations

The dynamic flow redistribution can significantly improve the small-world or scale-free public transit networks’ tolerance against random faults [43]. When a node fails, the flow it carries needs to be shared by other nodes connected to it. To quantitatively determine the proportion shared by neighboring nodes. Thus, the spare V/C ratio (SVR) is introduced to describe the capacity of the neighboring nodes to share the flow through the failed node. Theoretically, as long as the V/C ratio is not higher than 1, the traffic demand does not exceed the capacity, and traffic congestion does not occur. However, owing to the stochastic variations in traffic, traffic congestion might have already occurred when the V/C ratio is close to 1. The Webster Method, often called the TRRL Method, is one of the most commonly used methods in the process of traffic signal control design. The V/C ratio not exceeding 0.9 is a design constraint that must be met in this method [49, 50]. We adopt the same view and believe that a saturation level of no more than 0.9 is the upper limit for acceptable service levels. Therefore, we assume that the V/C ratio of the neighboring nodes cannot exceed 0.9 after sharing the detour traffic flow. If the V/C ratio of a neighboring node already exceeded 0.9 before the shared detour flow, it cannot share the flow anymore. Thus, the SVR of node *i* is defined as,

$$x_{Fi}=\frac{1}{m}\sum_{j=1}^{m}(0.9-x_{j}),\;\forall x_{j}=\{\begin{array}{cc} x_{j} & ,x_{j}<0.9\\ 0.9 & ,x_{j}\geq 0.9 \end{array},$$ (4)

where *m* is the number of neighboring nodes linked directly with node *i*, and *x*_*j*_ is the V/C ratio of neighboring node *j*.

**Fig 3:**
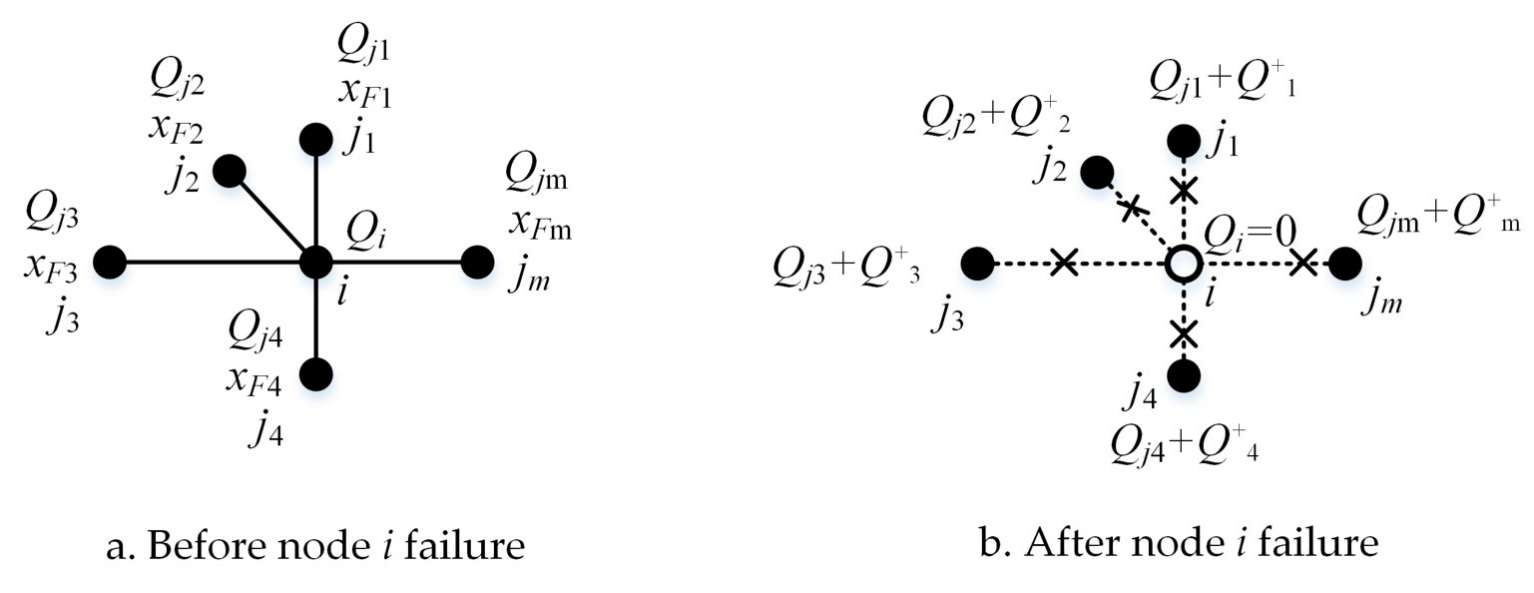
Rule of the local flow redistribution when node *i* fails.


The rule of flow redistribution is described in Fig 3. The initial traffic volume of the node *i* is *Q*_*i*_. There are *m* neighboring nodes connected with node *i*. The initial traffic volume of the node *j*_*m*_ is *Q*_*jm*_, and the SVR of the node *j*_*m*_ is *x*_*Fm*_ (Fig 3(A)). When the node *i* fails, *Q*_*i*_ is shared by all neighboring nodes connected with node *i*. The ability of neighboring nodes to share the detour flow is calculated by Eq (4). Their traffic volume will be changed, and the increased traffic volume of node *j*_*m*_ will be calculated according to Fig 3(B) and Eq (5).

$$Q_{m}^{+}=\frac{x_{Fj}}{\sum_{1}^{m}x_{Fj}}\cdot Q_{i},\;\forall j=1,2,\ldots ,m,$$ (5)

where $Q_{m}^{+}$ is the increased traffic volume of node *j*_*m*_. Thus, the traffic volume of node *j*_*m*_ after a disturbance will be changed to $Q_{jm}+Q_{m}^{+}$. When calculating the SVR of nodes connected to the failed node, the failed node is removed from the network.

#### 2.3.4. Cascading failures analysis

This study proposed an improved CML model (ICML) that considered the tunnel factors and the flow redistribution to analyze the cascading failures of the weighted freeway network. The ICML provides a theoretical basis for the accurate vulnerability analysis of the freeway network. The following equation expresses the model mathematically,

$$x_{i}(t+1)=|(1-\xi_{1}-\xi_{2})\cdot f(x_{i}(t))+\xi_{1}\cdot \sum_{j=1,j\neq i}^{N}TF_{ij}^{edge}\cdot \frac{f(x_{j}(t))}{TF_{i}^{node}}+\xi_{2}\cdot \sum_{j=1,j\neq i}^{N}w_{ij}\cdot \frac{f(x_{ij}(t))}{s(i)}|\;i=1,2,3\ldots N,$$ (6)

where *x*_*i*_(*t*) is the state variable of the node *i* at the *t*th time step. The adjacency matrix $A={\left(TF_{ij}^{edge}\right)}_{N\times N}$ represents the network’s topology based on the use of the tunnel factors as the weights of the edges. In this case, *w*_*ij*_ is the traffic flow between nodes *i* and *j*, and *s*(*i*) is the sum of flow in the edges connected with node *i*. In addition, *ξ*_1_ and *ξ*_2_ are defined based on the constraints such that *ξ*_1_,*ξ*_2_∈(0,1), and *ξ*_1_+*ξ*_2_ = 1. The function *f* defines the local dynamics chosen in this work as the chaotic logistic map, *f*(*x*) = 4*x*(1−*x*). Using the absolute value in Eq (6) guarantees that each node’s state is always nonnegative.

If the initial states of all nodes in the network are in the interval (0, 1) and there is no external perturbation, all the nodes will maintain a normal state forever (0<*x*_*i*_(*t*)<1, *t*≤*m*); if the flow of node *i* exceeds the capacity constraints at the *m*th time step (*x*_*i*_(*m*)≥1, *t*>*m*), then node *i* will fail at the *m*th time step and the state of the failed node will be assumed *x*_*i*_(*t*) = 0 at all subsequent time instants.

The freeway network encounters a sudden perturbation attributed to traffic accident variations, sudden traffic flow, or geological disasters, which lead to nodal failure or to the shutdown of a toll station. An external perturbation *R*≥1 to node *i* at the *m*th time step is added to show the attack effects. The modified model is expressed as follows,

$$x_{i}(t+1)=|(1-\xi_{1}-\xi_{2})\cdot f(x_{i}(t))+\xi_{1}\cdot \sum_{j=1,j\neq i}^{N}TF_{ij}^{edge}\cdot \frac{f(x_{j}(t))}{TF_{i}^{node}}+\xi_{2}\cdot \sum_{j=1,j\neq i}^{N}w_{ij}\cdot \frac{f(x_{ij}(t))}{s(i)}|+R$$ (7)

If the node *i* at the *m*th time step fails, the state is *x*_*i*_(*t*) = 0, *t*>*m*. At the (*m*+1)th time step, the states of those nodes directly connected with the node *i* are affected, and the flow is recalculated according to Fig 3 and Eqs (4) and (5). If a node’s state changes to greater than 1, then the node fails, which causes a cascading failure. Repeat the above steps until no more nodes fail or all nodes fail. The cascading failure proportion of the entire network at *t*th time step is defined as shown in Eq (8).

$$P(t)=\frac{N'(t)}{N}$$ (8)

where *N*’(*t*) is the number of failed nodes in the network at *t*th time step, and *N* is the number of nodes in the network. The proportion of cumulative nodal failure at each time step is used to characterize freeway cascading failure process.

In this process of a node being malicious attacked, two indicators can be captured. The first one is the perturbation threshold of cascade failure. The value of disturbance *R* added to this node in the ICML model is gradually increased until the cascade failure is triggered. The *R* value at this time is defined as the perturbation threshold of cascade failure of this node. The higher the *R* value, the less prone the node to trigger cascading failure. The *R* value of this node can be used to judge the probability of nodal vulnerability. The second one is the time step (*TS*) that records the duration from when the node is attacked to the complete failure of the entire network. The value of the *TS* indicates the propagation speed of cascade failure. The smaller the *TS*, the more serious the failure consequences. Thus, the *TS* can be used to judge the consequences of nodal vulnerability. All nodes of the freeway network are maliciously attacked one by one using the ICML model. The *R* and *TS* of each node can be captured and will be used in the next step of the vulnerability evaluation.

### 2.4. Vulnerability assessment of freeway network based on risk analysis

#### 2.4.1. Nodal vulnerability index (*NVI*)

According to the previous analysis, it is more reasonable for vulnerability assessment to consider both the probability of vulnerability occurrence and the vulnerability of consequences, and this study adopts the same view. The nodal vulnerability index (*NVI*) is established to measure the nodal vulnerability in two steps. Firstly, the probability and consequences of vulnerability is captured using network cascade failure analysis. Since the *R* value and nodal vulnerability are negatively correlated, the inverse of the *R* value is taken as the probability of nodal vulnerability. Similarly, since the *TS* value and nodal vulnerability are negatively correlated, the inverse of the *TS* value is taken as the consequence of nodal vulnerability. Secondly, the *NVI* is expressed as the product of consequence and probability based on risk analysis, as follows:

$$NVI_{i}=\frac{1}{R_{i}}\cdot \frac{1}{TS_{i}}$$ (9)

where *R*_*i*_ is the triggering threshold of cascade failure when node *i* is attacked. *TS*_*i*_ is the time step from when node *i* is attacked to the complete failure of the entire network. The larger the *NVI* of the node, the higher the vulnerability of the node.

#### 2.4.2. Vulnerability classification using a hierarchical cluster algorithm

All evaluated nodes can be grouped into clusters according to their *NVIs* to decide which nodal vulnerabilities are acceptable or unacceptable. To identify the vulnerability classification threshold, a hierarchical cluster algorithm is applied to allow for pairwise comparison through Euclidean distance between clusters [51]. Hierarchical clustering is a data-driven method, which can overcome subjective bias and has been widely applied in hierarchical rank studies [52, 53]. In the clustering process, every object is in its own cluster at the beginning and then sequentially combined into larger clusters according to similarity. We applied the hierarchical clustering method to group clusters step by step. The optimum cluster number with minimal variability is determined by the elbow method [54].

## 3. Case study

A real freeway network in Fuzhou of China is selected as an example to demonstrate the applicability of the proposed method.

### 3.1. Data preparation

#### 3.1.1. Freeway network

**Fig 4:**
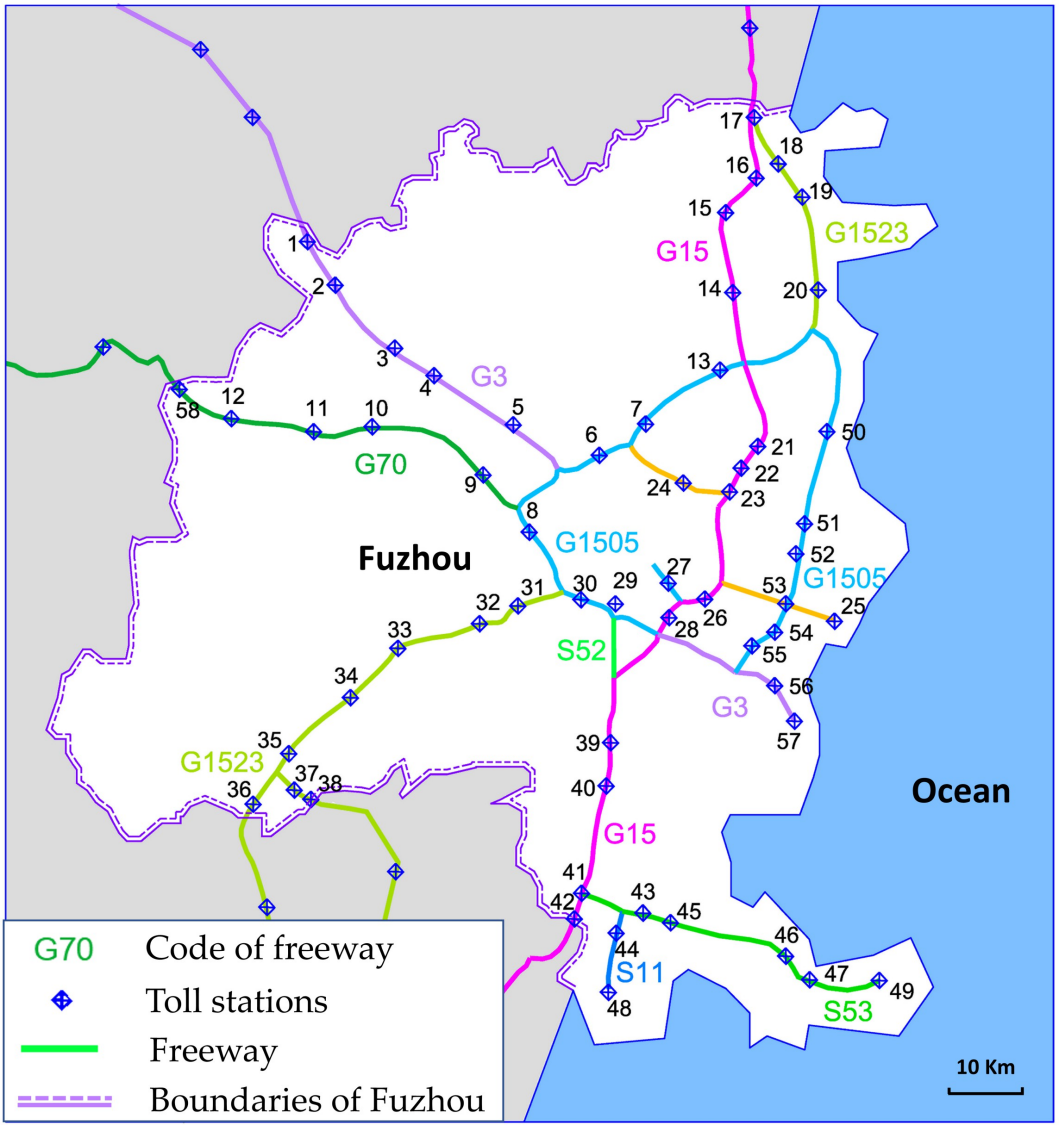
Freeway network of case study.

Our case area selected for this study included the Fuzhou freeway networks (FFN) located in Fujian Province, China. Fuzhou, the Fujian Province’s capital, is one of the important cities along the southeast coast of China. Fuzhou has a total land area of 11,968 km^2^, of which the urban area occupies 1,786 km^2^, with a well-developed transportation network. By the end of 2019, the total highway mileage was 11,659 km, and the total freeway mileage reached 673 km [55]. There were nine freeways in the FFN and one freeway was marked with one particular color (Fig 4). In these freeways, six freeway routes extend beyond the boundaries of Fuzhou. There were 58 toll stations in the FFN.

#### 3.1.2. Flow and volume-to-capacity ratio

**Fig 5:**
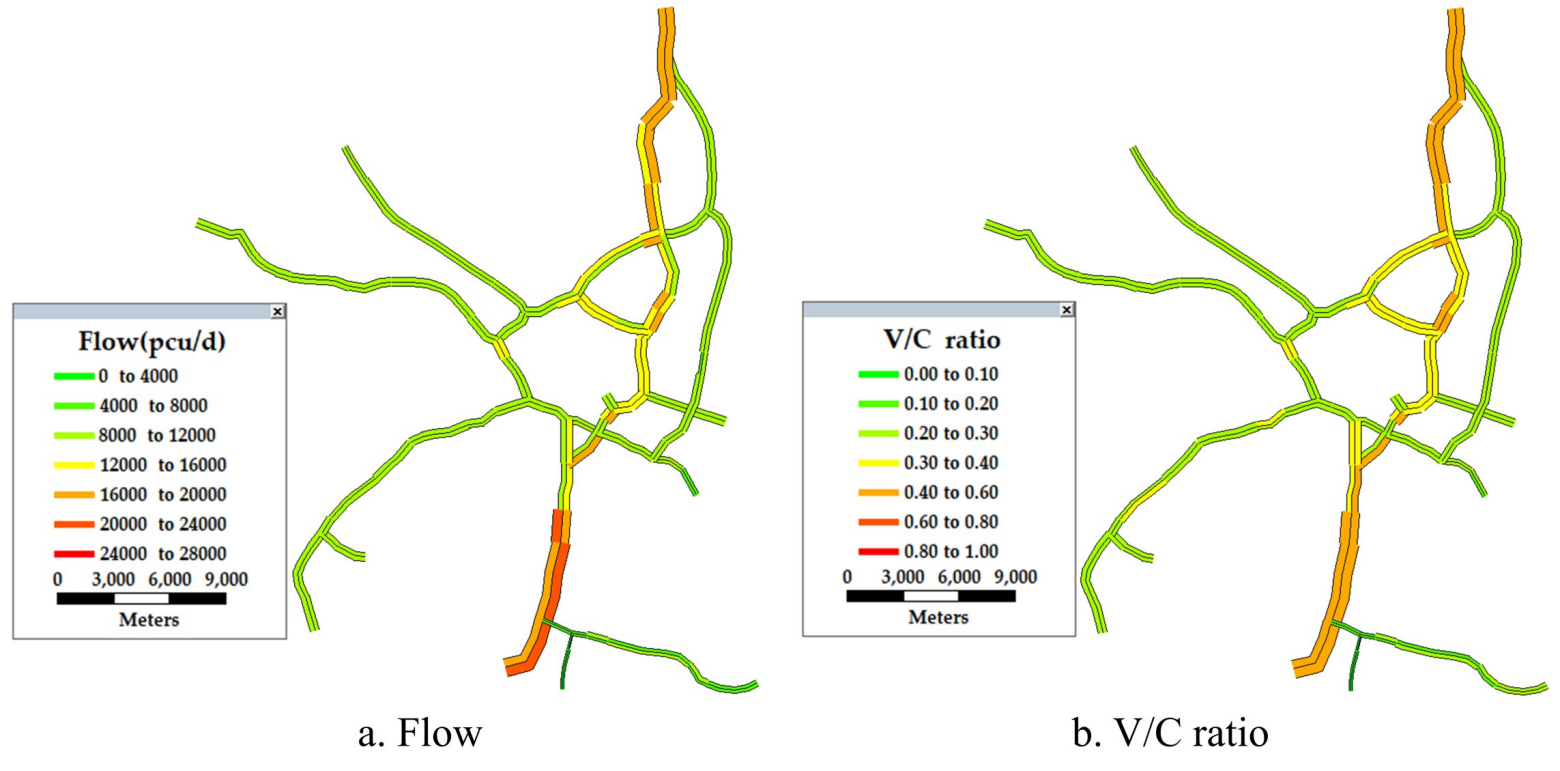
Flow and V/C ratio of the freeway in the FFN.

The daily traffic flow and volume-to-capacity ratio (V/C ratio) of the freeways in the research area were obtained from the Fujian Provincial Freeway Information Technology Co., Ltd. (see Fig 5).

#### 3.1.3. Tunnel data

Fujian is a mountainous province. Mountains and hills account for 72.68% of the total land area in the region. Therefore, the number of highway tunnels ranks in the top five of the country. The longest highway tunnel in Fujian is within the Fuzhou–Niuyanshan Tunnel and spans 9,252 m. According to the Specifications for Design of Highway Tunnels Section 1 Civil Engineering (JTG 3370.1–2018), tunnels can be classified in four types: extra-long, long, medium, and short tunnels based on their lengths, as shown in Table 1.

**Table 1:** Tunnel length classification.

Tunnel classification	Extra-long tunnel	Long tunnel	Medium tunnel	Short tunnel
**Length (m)**	L > 3000	3000 ≥ L > 1000	1000≥ L> 500	L ≤ 500

In the FFN, there are 82 tunnels with a total length of 23.57 km, including 8 extra-long tunnels, 30 long tunnels, 15 medium tunnels, and 29 short tunnels. Owing to space limitations, only the specific data of the extra-long tunnel are listed in Table 2.

**Table 2:** Eight extra-long tunnels in the FFN.

No.	Freeway code	Freeway name	Tunnel name	Tunnel length (m)
1	G3	Jingtai Freeway	Niuyanshan Tunnel	9,252
2	G3	Jingtai Freeway	Tianlongshan Tunnel	6,551
3	G1523	Yongguan Freeway	Youcheling Tunnel	5,754
4	G70	Fuying Freeway	Meigulin Tunnel	5,580
5	G1505	Fuzhou Ring Freeway	Guixin Tunnel	4,928
6	G3	Jingtai Freeway	Fendongshan Tunnel	4,911
7	G1523	Yongguan Freeway	Menqianshan Tunnel	3,656
8	G15	Shenhai Freeway	Feiluanling Tunnel	3,180

**Fig 6:**
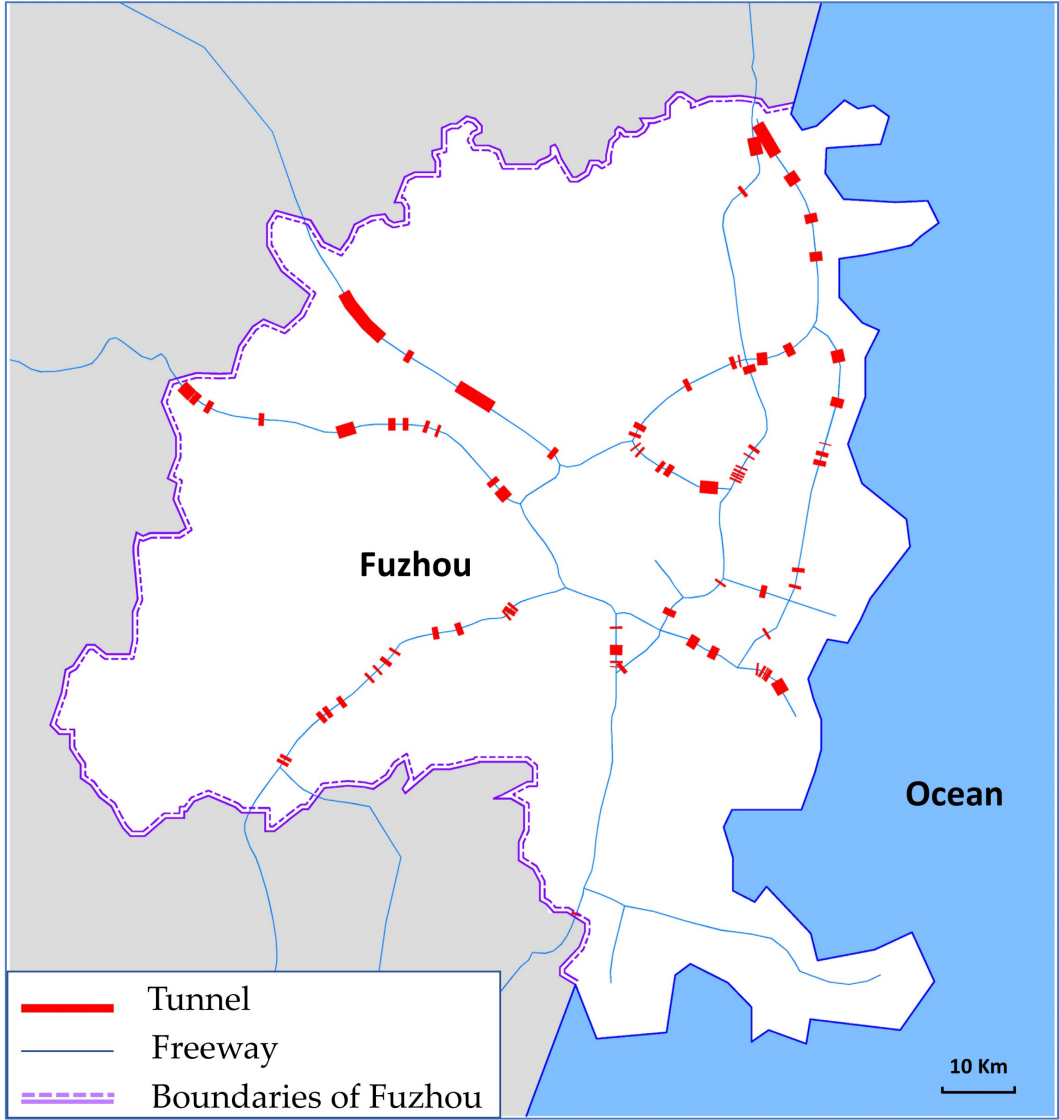
Location and length of the tunnels in the FFN.

Fig 6 shows the locations and lengths of all tunnels in the FFN. The lengths of the red thick lines represent the lengths of the tunnel.

### 3.2. Complexity analysis of FFN

The complexity of the freeway network will affect its operational efficiency. The complexity indicators (i.e., degree, betweenness, and average path length [56, 57]) are used to analyze the freeway network’s complexity. As mentioned in section 2.2, we introduce virtual peripheral nodes to eliminate the boundary effects that cause errors in topology and traffic redistribution. Because virtual peripheral nodes are beyond the scope of this research, they are not shown in this section, although they are considered in the analysis.

#### 3.2.1. Topological representations

**Fig 7:**
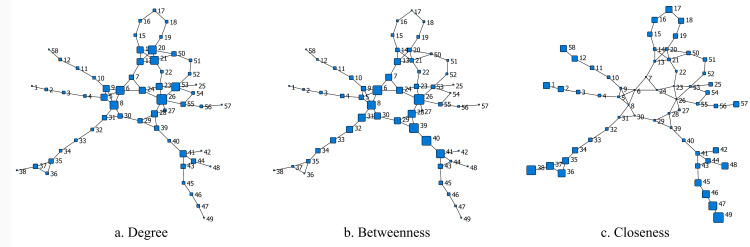
Topological graph of the FFN associated with different indicators.

Based on CNT [58, 59], the adjacency matrix of networks was constructed in terms of the connective relationships of freeways. According to the rules mentioned in section 2.2., the FFN can be represented to a topological graph with 58 nodes with 154 directed links, including four endpoint nodes whose degrees is equal to one. Since six freeway routes extending beyond the FFN, there are six additional virtual peripheral nodes. In the topological graph, each nodal size denotes special indicator values (Fig 7). The adjacency matrix was then imported and analyzed with the use of the network analysis software programs UCINET and NETDRAW [60–63].

#### 3.2.2. Degree and degree distribution

**Fig 8:**
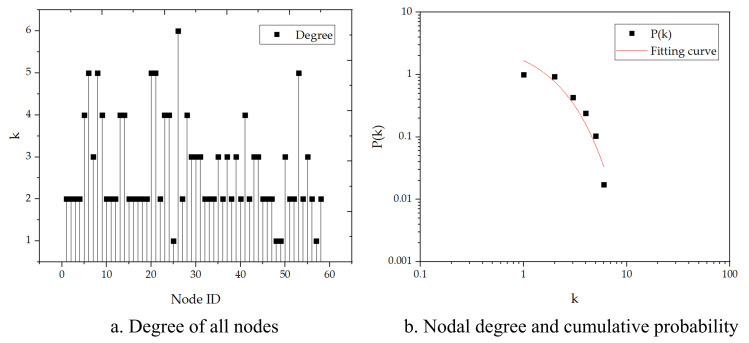
Characteristics of degree distribution in the FFN.

The nodal degree is the most straightforward index used to quantify the individual centrality. It is believed that the most critical node must be the most active one [58]. Statistical results showed that the average degree was 2.724 of the FFN. Approximately 57% of the FFN’s nodes had a degree which was not more than two (Fig 8(A)). The characteristic that more than half of the nodes have a degree of two implies that once a perturbation occurs in the network, cascade failures are more likely to be triggered and propagate more quickly through the network. The nodal degree and the distribution of the FFN’s cumulative degree were fitted in the double logarithmic coordinate system, as shown in Fig 8(B). The plot of the cumulative degree does not follow a power-law distribution, thus indicating that the FFN is not a scale-free, heterogeneous network [64].

#### 3.2.3. Average path length

**Fig 9:**
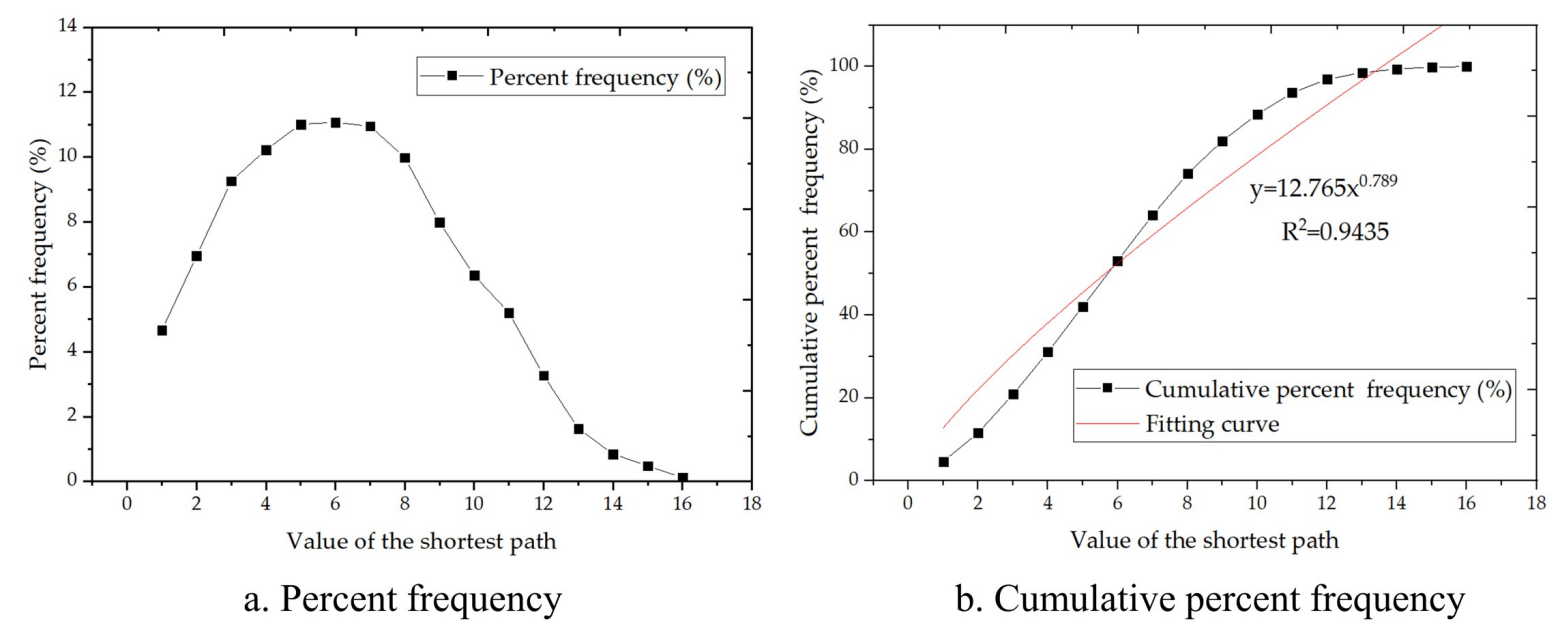
Characteristics of the shortest path distribution in the FFN.

The average path length is a global property important to communication in networks, directly reflecting the reachability of the entire freeway network. The characteristic path length is the average of all pairwise shortest-path lengths between nodes in the network. The maximal value of the shortest path is the diameter of the network. Therefore, the FFN’s reachability is judged by comparing the values of the shortest path between any two nodes with the size of the network. The characteristics of the shortest path distribution in the FFN are shown in Fig 9. The distribution of the percent frequency of the shortest path follows the normal distribution (Fig 9(A)). Fig 9(B) shows the corresponding cumulative frequency of each shortest path between any nodes in the FFN. The average shortest path length for a user between any toll stations is 6.18, which is smaller than the size of the network 16. Therefore, the FFN possesses relatively good reachability in the current research.

#### 3.2.4. Node ranking based on different indicators

Some parameters, such as the nodal degree, betweenness and initial state, can effectively describe the importance of specific nodes. In this study, the V/C ratio was selected as the indicator of the initial state of the node. The degree, betweenness, and initial state of each node in the FFN were calculated first, and the top five nodes were listed in Table 3. Node 26 is the node with the largest degree of six, which means that it connects with the other six toll stations within the network. Node 39 possesses the largest node betweenness of 572, indicating that 572 shortest paths within the network pass it. Node 40 is the node with the highest initial state of 0.6851, which means that it is under maximum traffic pressure in all toll stations. It is also worth noting that the three rankings are quite different.

**Table 3:** Top five most important primary nodes ranked among different indicators in the FFN.

Based on degree			Based on betweenness			Based on the initial state		
Rank	Node ID	*k*	Rank	Node ID	*c*_*Bi*_	Rank	Node ID	*x*_*i*_(*t*_0_)
1	26	6	1	39	572	1	40	0.6851
2	6	5	2	26	570.667	2	15	0.6784
3	8	5	3	6	552.167	3	16	0.6769
4	20	5	4	8	533.667	4	13	0.5656
5	21	5	5	40	530	5	14	0.5598

### 3.3. Vulnerability assessment of FFN

#### 3.3.1. Cascade failure analysis

**Fig 10:**
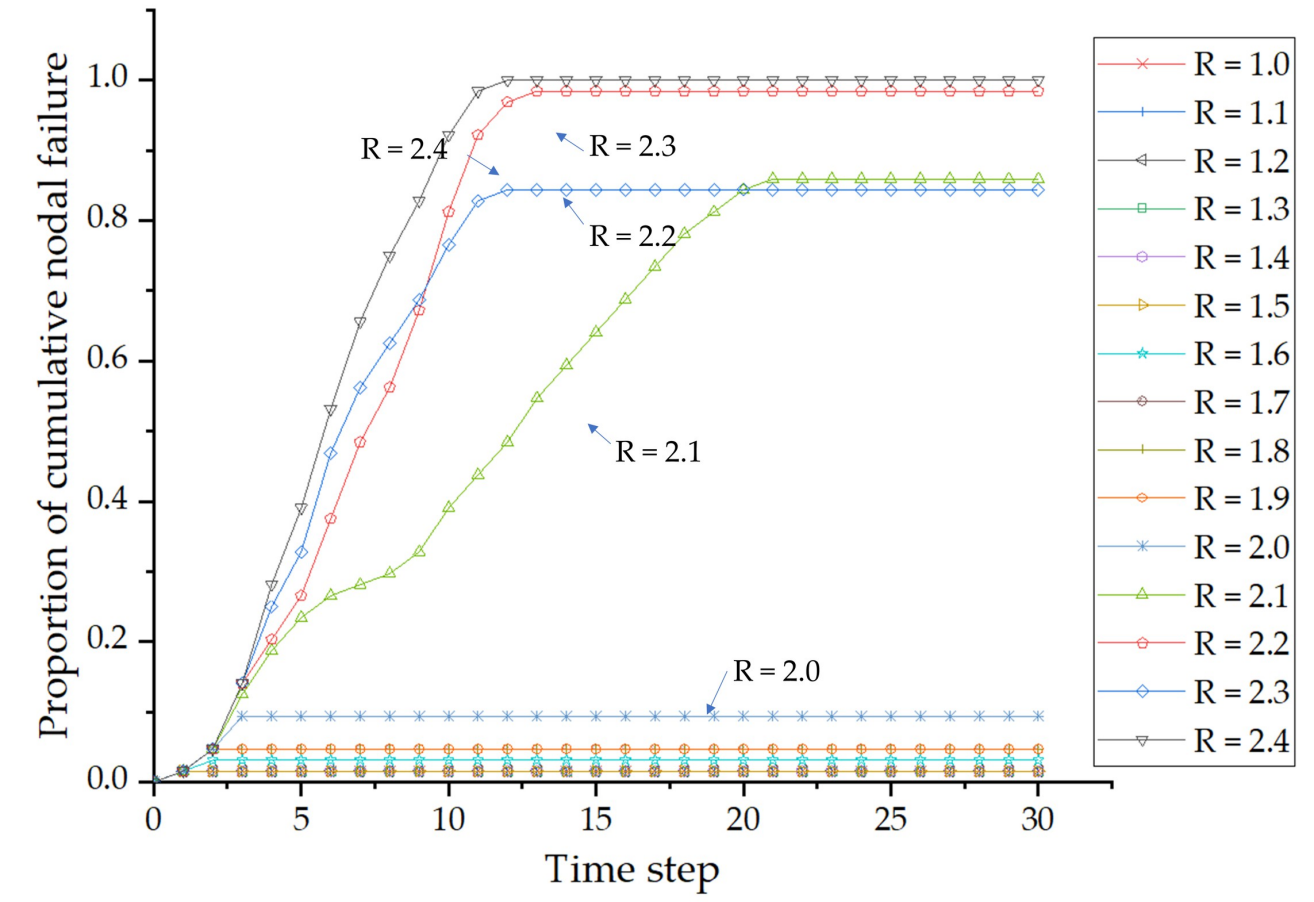
Spreading process of cascading failure at different perturbations (node 4).

We take node 4 as an example to illustrate the process of nodal cascade failure analysis based on the ICML model. When node 4 is under attack, the spreading process of cascade failure as the perturbation gradually increases is shown in Fig 10.

As shown in Fig 10, when the perturbation *R* increases from 1.1 to 2.0, the proportion of cumulative nodal failure is at most 10%, and cascade failure is not triggered. When the perturbation increases to *R* = 2.1, cascading failures are triggered. The cascade failure lasted for 22 time steps, and the cumulative percentage of failed nodes reached 85.9%. Then, the remaining nodes ceased to fail. When *R* = 2.4 is added to node 4, cascading failures propagate throughout the network. The cascade failure lasted for 12 time steps, and the cumulative percentage of failed nodes reached 100%. The perturbation value *R* = 2.4 that triggers failure of the entire network is used as the perturbation threshold of node 4 to calculate the probability of nodal vulnerability. *TS* = 12 is used to calculate the probability of nodal vulnerability.

**Fig 11:**
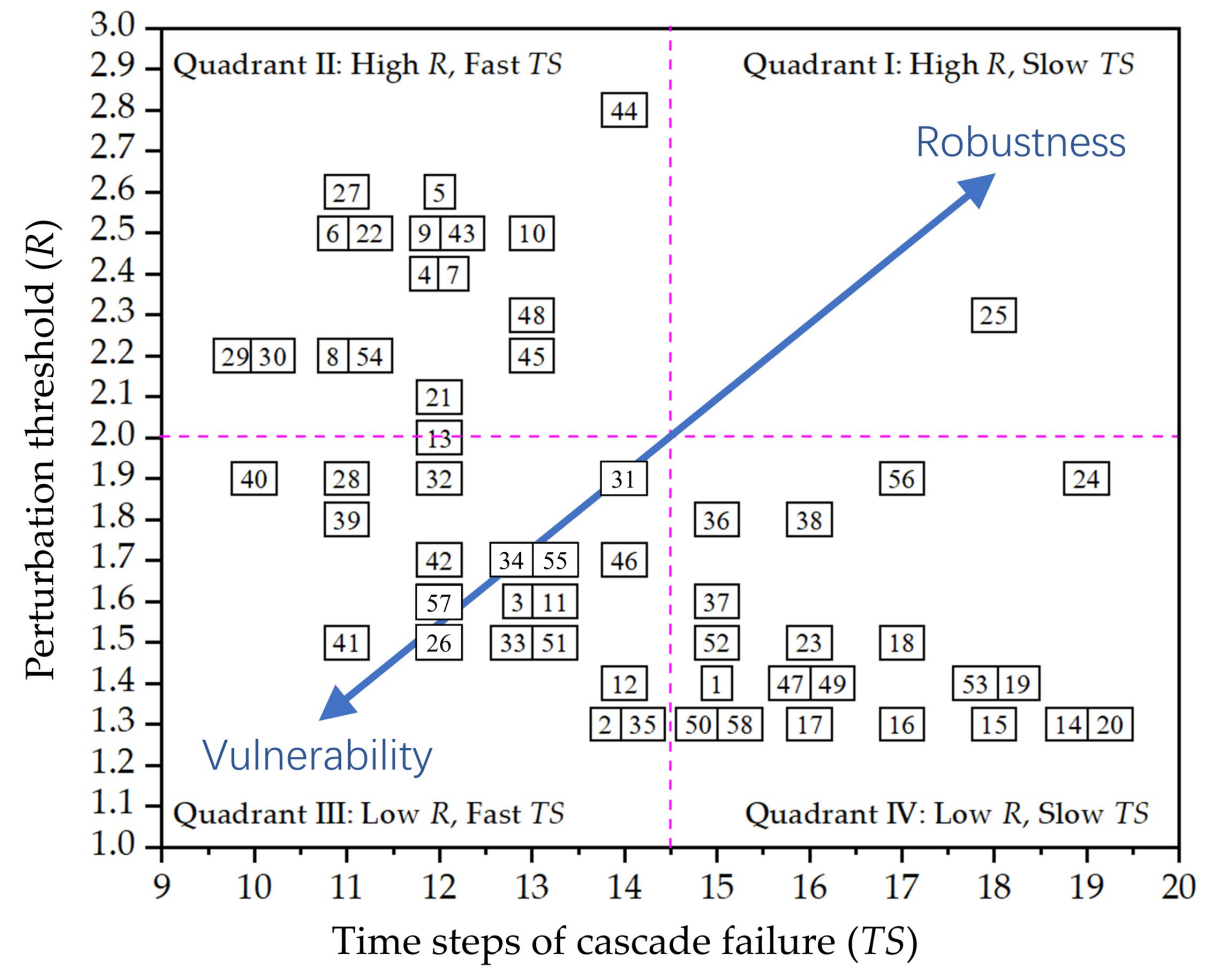
The perturbation threshold and propagation speed of cascade failure of each node.

All nodes of the FFN are maliciously attacked one by one, and the cascade failure results of all nodes in the FFN are shown as a risk matrix [11] in Fig 11.

In Fig 11, the x-axis represents the propagation time step of the cascade failure *TS*, and the y-axis represents the disturbance threshold *R* when the cascade failure is triggered. Based on the idea of the four-quadrant method, the two dotted lines in Fig 11 divide the coordinate system into four quadrants. The different quadrants indicate the different performances of the freeways during cascading failures. The nodes in quadrant I that have higher *R* and larger *TS* have a lower probability of vulnerability and less severe vulnerability consequences. These nodes have stronger robustness as well. On the contrary, the nodes in quadrant III have lower *R* and smaller *TS*, indicating that these nodes are more vulnerable than the nodes in the other three quadrants. The blue line with two arrowheads in Fig 11 indicates two opposite changing trends in node vulnerability, with nodes closer to the upper right corner of the coordinate system being more robust and those closer to the lower left corner of the coordinate system being more vulnerable. The vulnerability of some nodes can be observed schematically based on the spatial location of the nodes in Fig 11. However, it is difficult to obtain the vulnerability ranking of all nodes directly from Fig 11. For example, identification of nodes in quadrant II and quadrant IV that are more vulnerable to attack requires further analyses.

#### 3.3.2. *NVI*s in FFN

After finding the perturbation threshold and the time step of cascade failure propagation of each node, the *NVI* of all nodes in the network can be obtained using Eq (9). The nodes in the FFN are ranked according to their *NVI* values, as listed in Table 4.

**Table 4:** *NVI*s in FFN.

Rank	Node ID	Node name	*NVI*	Rank	Node ID	Node name	*NVI*
1	41	Yuxi	0.0606	31	23	Mawei	0.0417
2	26	Yingqian	0.0556	32	37	Youyang	0.0417
3	2	Yangli	0.0549	33	8	Fuzhouxi	0.0413
4	35	Wutong	0.0549	34	54	Zhanggang	0.0413
5	40	Honglu	0.0526	35	14	Danyang	0.0405
6	57	Songxia	0.0521	36	20	Pukou	0.0405
7	33	Yongtaidong	0.0513	37	19	Mabi	0.0397
8	50	Dingan	0.0513	38	21	Lianjiang	0.0397
9	58	Yangzhong	0.0513	39	53	Jiangzhu	0.0397
10	12	Jinsha	0.0510	40	18	Luoyuanwan	0.0392
11	39	Jingyang	0.0505	41	31	Qishan	0.0376
12	42	Hanjiang	0.0490	42	36	Caixi	0.0370
13	3	Dahu	0.0481	43	6	Jingxi	0.0364
14	11	Minqing	0.0481	44	22	Guantou	0.0364
15	17	Feiluan	0.0481	45	27	Fuzhou	0.0350
16	51	Langqi	0.0481	46	45	Gangtou	0.0350
17	28	Huangshi	0.0478	47	4	Baisha	0.0347
18	1	Dongqiao	0.0476	48	7	Guihu	0.0347
19	29	Lanpu	0.0455	49	38	Zhuangbian	0.0347
20	30	Xiangqian	0.0455	50	48	Jiangkoudong	0.0334
21	16	Luoyuan	0.0452	51	9	Minhou	0.0333
22	34	Yongtaixi	0.0452	52	43	Jiangjing	0.0333
23	55	Yutian	0.0452	53	5	Ganzhe	0.0321
24	47	Pingtan	0.0446	54	56	Binhai	0.0310
25	49	Pingtandong	0.0446	55	10	Meixi	0.0308
26	52	Tantou	0.0444	56	24	Kuaian	0.0277
27	32	Geling	0.0439	57	44	Jiangyin	0.0255
28	15	Luoyuanna	0.0427	58	25	Fuzhoujichang	0.0242
29	46	Gaoshan	0.0420				
30	13	Guian	0.0417				

From the results in Table 4, the node with the largest *NVI* is 41, indicating that the toll station Yuxi is the most vulnerable. The top five vulnerable nodes are 41, 26, 2, 35, and 40. Conversely, node 25 has the smallest *NVI*, indicating that it is the most robust. The vulnerability characteristics of these nodes coincide with the results observed in Fig 10. It is also worth noting that the *NVI* rankings are quite different from the degree rankings and betweenness rankings (Table 3).

#### 3.3.3. Identification of critical nodes

To identify the critical nodes, all evaluated nodes in the FFN were grouped into clusters according to their *NVIs* using a hierarchical cluster algorithm. The optimum cluster number with minimal variability is five, as determined by the elbow method. The measurements of *NVI* levels are listed in Table 5.

**Table 5:** Thresholds for these levels of the nodal vulnerability.

Clusters	Score	Rank
1	0.0549–0.0606	Extremely high
2	0.0476–0.0526	High
3	0.0392–0.0455	Medium
4	0.0308–0.0376	Low
5	0.0242–0.0277	Extremely low

**Fig 12:**
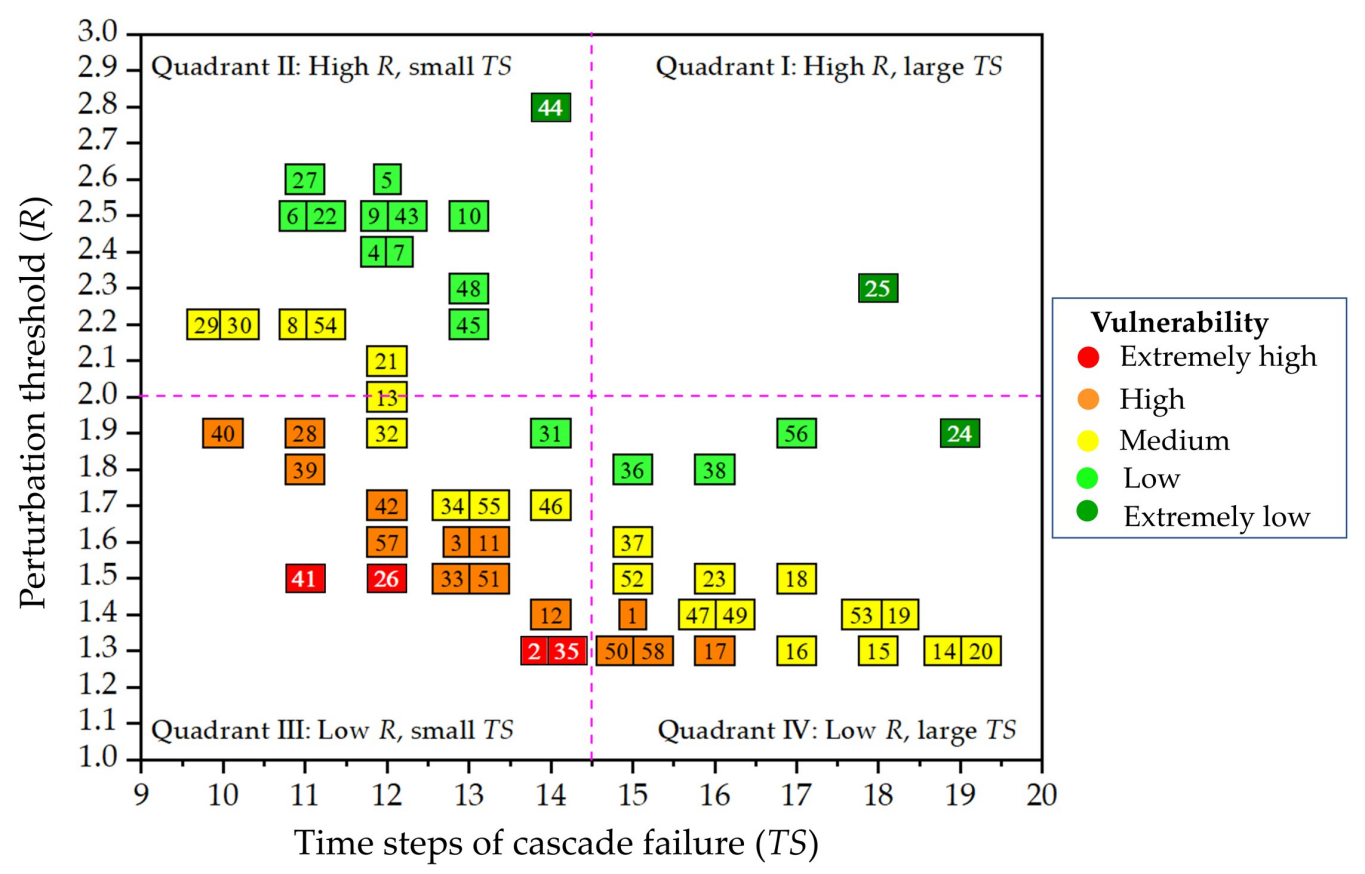
Results of hierarchical clustering of the nodal vulnerability.

The results of the hierarchical clustering are visualized in a four-quadrant coordinate system, as shown in Fig 12. It can be seen that there are four nodes with extremely high vulnerability, i.e., nodes 41, 26, 2, and 35, accounting for 6.9% of all nodes. There are 14 nodes with high vulnerability (24.1%), 22 nodes with medium vulnerability (37.9%), 15 nodes with low vulnerability (25.9%), and three nodes with extremely low vulnerability (5.2%).

**Fig 13:**
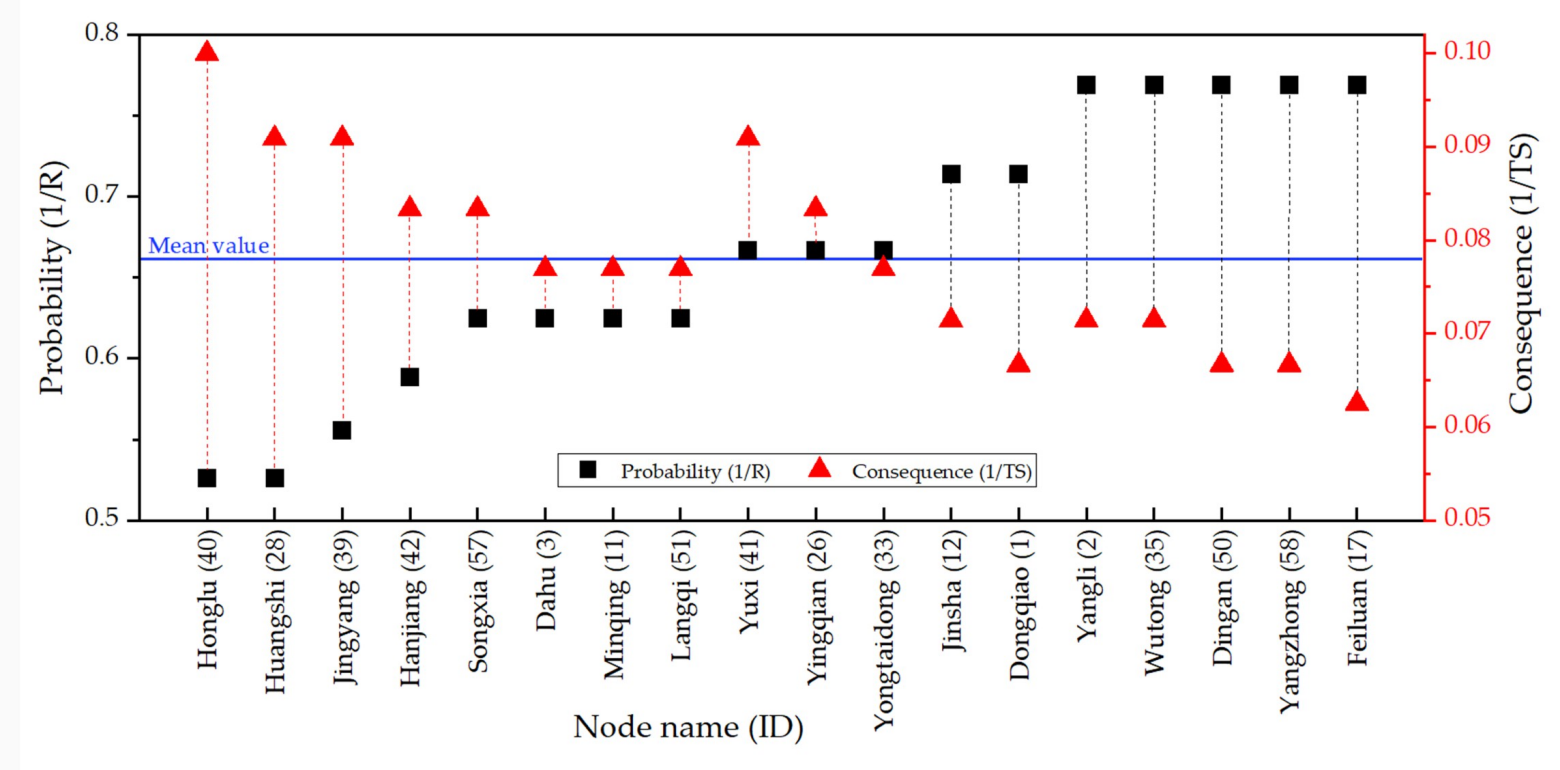
The comparison of probability and consequences of nodal vulnerability.

There are 18 nodes with extremely high or high vulnerability in the FFN. These nodes require more attention from road administrators. Fig 13 illustrates comparisons between the probabilities and consequences of nodal vulnerability for the 18 nodes in the FFN, indicating that the probability of nodal vulnerability does not always equate to consequence. Honglu (node 40), for example, was ranked first for the consequence of nodal vulnerability but had a lower probability ranking, indicating that the probability of a closure occurring at Honglu is extremely low, but when it does, the consequences are more severe and considerably larger in number than those at other toll stations. The blue horizontal line in Fig 13 represents the average value of probability and consequence. It is clear from the graph that the probability or consequence of each node is higher or lower than the average. Among the high-consequence and low-probability toll stations, the gap between consequence and probability was the largest for Honglu, Huangshi, Jingyang, and Hanjiang. On the contrary, among the high-probability and low-consequence toll stations, the gap between consequence and probability was the largest for Feiluan, Yangzhong, Dingan, Wutong, Yangli, and Dongqiao. For nodes with high probability or high consequence, the road administrators should respond with two distinct strategies, one aimed at reducing the probability of a disruptive event occurring and the second aimed at reducing the consequences of a disruptive event that occurs. For nodes with a high probability of vulnerability, some pre-emptive measures should be taken to minimize the probability of disruptive events. For example, traffic flow is monitored in real time, and when certain warning values are reached, measures to limit the flow are taken in advance. Similarly, for nodes with serious vulnerability consequences, some mitigative measures should be carried out to mitigate the consequence of node failure. These measures include improving the capacity for accident rescue and obstructed road clearance as well as pre-planning for traffic detours.

**Fig 14:**
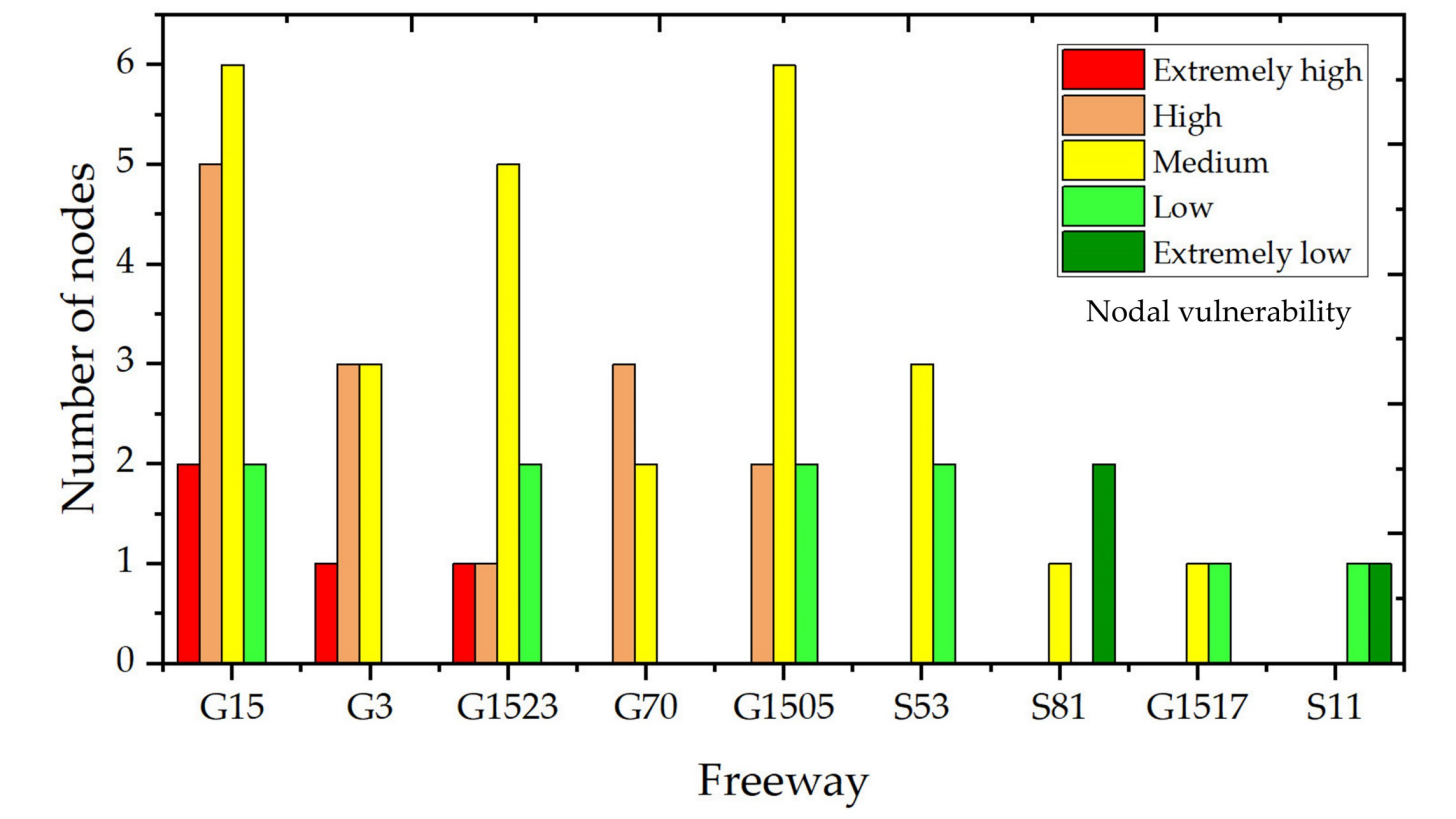
Number of nodes within different vulnerability clusters on each freeway.

Since the maintenance work of each freeway is assigned to different maintenance companies, it is necessary to conduct a vulnerability analysis for each freeway in order to make corresponding improvement suggestions for each maintenance company. The number of nodes of different vulnerability clusters on each freeway in the FFN is counted and arranged in descending order, as shown in Fig 14.

According to Fig 14, among the 15 toll stations along G15, 46.7% of them have extremely high or high nodal vulnerability. In G15, there are two toll stations with extremely high vulnerability, namely Yuxi and Yingqian, and five toll stations with high vulnerability, namely Feiluan, Huangshi, Jingyang, Honglu, and Hanjiang. G15 (Shenhai Freeway) is the longest freeway in the FFN and runs through the whole of Fuzhou, connecting the north and the south. Thus, G15 is an extremely important freeway, but it is also the most vulnerable. Both G3 (Jingtai Freeway) and G1523 (Yongguan Freeway) have one toll station with extremely high vulnerability. Highways that contain toll stations with extremely high or high vulnerability include G15, G3, G1523, G70, and G1505. These freeways require more response measures from road administrators.

**Fig 15:**
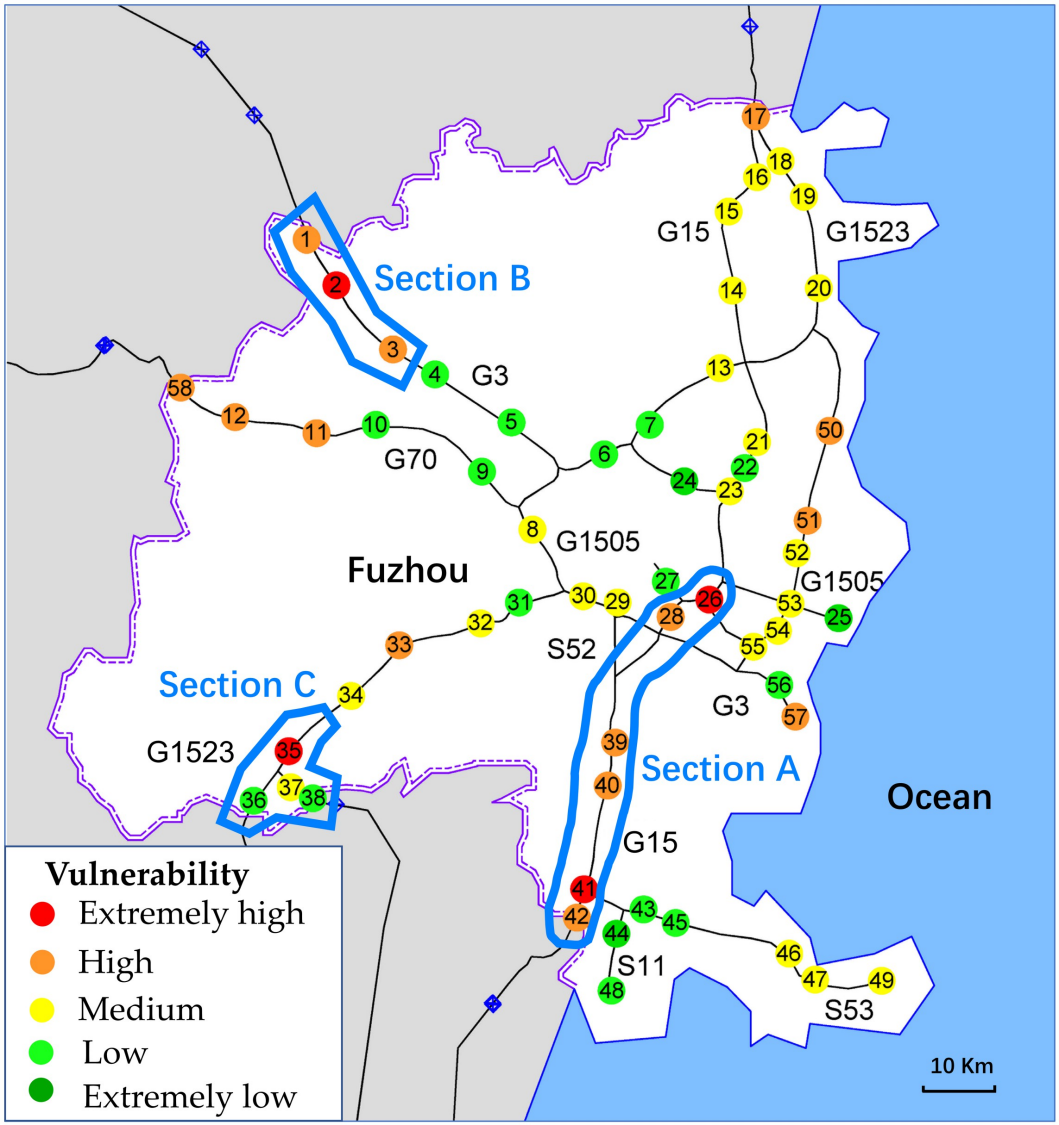
Spatial distribution of the nodal vulnerability in the FFN.

Fig 15 visualizes the spatial distribution of the nodal vulnerability in the FFN. In Fig 15, the three freeway sections that require the attention of road administrators are marked with blue lines, namely Section A, Section B, and Section C. Section A is the southern section of G15. There are six toll stations in this section, of which two have extremely high vulnerability, and four have high vulnerability, making it the most vulnerable section in the FFN. The main reason for the high vulnerability of this section is the extremely high volume of traffic it carries, which can easily trigger a failure when a minor accident occurs. Section B belongs to G3 and has one toll station with extremely high vulnerability and two toll stations with high vulnerability. The longest tunnel in Fuzhou is located in Section B, and the operational risk caused by the extra-long tunnel is the main reason for the high vulnerability of this section. Section C is part of G1523 and has only one node with extremely high vulnerability. This section has several short tunnel clusters and does not have much heavy traffic. The main reason for the high vulnerability may be that this toll station has a high topological risk due to the two virtual peripheral nodes connected to it from two directions.

Therefore, road administrators should take different measures to deal with different vulnerability risks. Section A should be the most critical focus of operational monitoring in the entire FFN, and it is essential to enhance real-time monitoring of this section to detect potential congestion early, so as to control traffic flow and prevent failures in advance. As for Section B, the operational stability in the extra-long tunnel should be enhanced by improving the safety facilities and traffic diversion via preplanning. For Section C, the construction of bypass freeways should be added to the next stage of the road network planning to improve its topological robustness.

## 4. Conclusions

Assessing the vulnerabilities and identifying the vital parts of a road network are critical tasks, as they provide road administrators with necessary information to maintain the network performance in the event of sudden disruptions. A framework to estimate the vulnerability of a freeway network and identify its critical parts based on risk analysis is presented herein. The nodal vulnerability index is defined as the product of the consequence and probability of perturbation, as in the definition of risk. Unlike previous risk analyses, the determination of the probability of occurrence and consequence of perturbations in this study is based on a novel perspective of network cascading failure analysis. A malicious attack is performed on a node and the severity of the perturbation is gradually increased until a cascade failure is triggered at a specific value. This particular perturbation value is used to indicate the probability that the node is vulnerable. In addition, the speed of propagation of the cascade failure in the road network is used to indicate the consequence of the vulnerability, as the faster the cascade failure propagates, the more severe is the consequence. In the process of cascading failure analysis, two main improvements were implemented in the ICML model. First, the degree in the topological coupling coefficient was replaced by the tunnel factor related to the length of the tunnel, which incorporated the negative effect of the freeway tunnels in the model. Second, the flow coupling coefficient based on the local flow dynamic redistribution was added to the model. The proposed algorithm for the local flow dynamic redistribution is practical and significantly reduces the computational burden. This method can considerably reduce the amount of required data and provides a new approach for risk analysis, which can be applied to other types of networks where cascading failures might occur.

In terms of network topological representation, this study improves upon the existing topological mapping methods by adding virtual peripheral nodes. In large-scale networks such as highway networks, the scale is often much larger than the scope of the study. Existing studies tend to remove all nodes and edges outside the study area based on the boundary of the study area. Thus, errors in topology analysis and traffic distribution occur at nodes near the boundary that have routes extending to nodes outside the study boundary, which leads to negative boundary effects. The improvement of the topological mapping rules in this study allows those nodes near the boundary to maintain the real topological relationship and traffic connectivity and eliminates the boundary effect.

The proposed methodology was applied to a real freeway network in Fuzhou, China. The results showed the following: 1) the FFN is not a scale-free, heterogeneous network; 2) the average degree of the FFN was 2.724. Approximately 57% of the nodes of the FFN had degree of not more than two; 3) the FFN possesses relatively good reachability, as the average shortest path length is smaller than the size of the network; 4) four nodes with extremely high vulnerability are the nodes 41, 26, 2, and 35, accounting for 6.9% of all nodes; 5) there are 18 nodes with extremely high or high vulnerability in the FFN, and their probability of nodal vulnerability does not always have a direct correspondence with the consequence; 6) road managers need to pay more attention to the three vulnerable highway sections (belonging to G15, G3, and G1523) and adopt different measures to address different vulnerability risks, such as enhance real-time monitoring to detect potential congestion early, improve the safety facilities in the extra-long tunnel to enhance the operational stability, and preplan bypass routes to improve the topological robustness. The case study demonstrates that the proposed framework is relatively simple but provides a reliable strategy to identify vulnerable areas and determine the corresponding response strategies for road administrators.

In terms of application prospects, this approach can be adapted to not only freeway networks but also other transportation networks with appropriate modifications to the CML model. As an example, in the case of a metro network, when a station is severely degraded due to heavy traffic, it may advisable to introduce the queuing theory to optimize the traffic redistribution algorithm in the CML model. Because different traffic networks have different congestion propagation characteristics, the methodology of this study can be applied to other transport networks after a more targeted calibration of the coupling coefficients of the CML model.

Although the method meets the requirements of road administrators for decision making, there are still some limitations that need further exploration in the future. For example, the method of local flow redistribution was preliminary, and we assumed that the redistribution of traffic flows only occurs at those nodes that are directly connected to the failed node. It is necessary to further optimize the rules of traffic redistribution in the local road network. Furthermore, only the scenario of a single node being attacked is analyzed in the cascade failure analysis, ignoring the complex scenario of simultaneous failures at multiple nodes.

## Supporting information

S1 File (ZIP)
